# Suppression of miR-130a-3p Attenuates Oxygen–Glucose Deprivation/Reoxygenation-Induced Dendritic Spine Loss by Promoting APP

**DOI:** 10.3389/fnins.2021.601850

**Published:** 2021-08-03

**Authors:** Liang Zhu, Lei Zhu, Jinyun Tan, Kui Chen, Bo Yu

**Affiliations:** ^1^Department of Vascular Surgery, Shanghai Pudong Hospital, Fudan University Pudong Medical Center, Shanghai, China; ^2^Department of Vascular Surgery, Huashan Hospital, Fudan University, Shanghai, China; ^3^Department of Neurology, Shanghai East Hospital, Tongji University School of Medicine, Shanghai, China

**Keywords:** cerebral ischemia, miR-130a-3p, dendritic spine, amyloid precursor protein, oxygen–glucose deprivation/reoxygenation

## Abstract

**Background:**

Cerebral stroke induces neuronal dysfunction as a consequence of neuronal morphology changes. Emerging evidence suggests that microRNAs (miRNAs) may play an important role in regulating dysfunction in stroke, yet there are still few studies examining the association between whole blood miRNAs and neuronal morphology. The present study aimed to ascertain the potential roles and mechanisms of action of miR-130a-3p in ischemic stroke.

**Methods:**

The miRNA datasets of peripheral serum in the GEO database and the mRNA datasets of the human brain after ischemia were analyzed to identify differentially expressed RNAs, and their functions were verified in cultured neurons *in vitro*. Furthermore, the target gene was validated by dual-luciferase reporter assay, RT-PCR, Western blot, and immunofluorescence experiments. The identified miRNA was further verified by the OGD test to restore neuronal changes after ischemia through APP.

**Results:**

The expression of whole blood miR-130a-3p was found significantly lower in participants with ischemic stroke than in controls by analyzing expression profiling datasets of cerebral ischemia stroke obtained from the Gene Expression Omnibus (GEO) DataSets portal, which was confirmed in the MCAO model in mice. Furthermore, GO analysis showed that miR-130a-3p might directly affect neuronal function. Indeed, we demonstrated that miR-130a-3p played a central role in the inhibition of dendritic morphogenesis and in the growth of dendritic spines *in vitro*. We also confirmed that miR-130a-3p could regulate the expression of *APP* by luciferase reporter assay, RT-PCR, Western blot, and immunofluorescence experiments, which were consistent with the bioinformatic analysis. Last but not least, we also demonstrated that reducing miR-130a-3p expression partially rescued neuronal morphological changes after OGD *in vitro*.

**Conclusion:**

miR-130a-3p is a potential biomarker of cerebral stroke, can affect neuronal morphology through *APP*, and promote the repair of neurons by promoting *APP* expression after cerebral ischemia.

## Introduction

Cerebral ischemia (CI) is one of the most common diseases of the central nervous system (Diener and Hankey, 2020), accounting for approximately 10% of all deaths globally in 2016 (Feigin and Vos, 2019). Ischemic stroke represents one of the leading causes of death worldwide (Katan and Luft, 2018) and is estimated to be the fourth most common cause of increased disability-adjusted life years by 2030 (Donnan et al., 2008), causing long-lasting disabilities and thereby contributing to a high socioeconomic burden (Feigin and Vos, 2019). Despite considerable efforts in preclinical and clinical research, stroke therapy is still limited due to unclear pathology (Khoshnam et al., 2017). In this process, changes in dendrites and dendritic spines are important factors (Zhu et al., 2017). Stroke induces rapid neuronal deterioration, including spinal loss and longer survival of dendritic spines in the peri-infarct cortex (Zhao and Willing, 2018). These morphological changes decrease the density of synapses and induce neurologic impairments (Brown et al., 2008).

microRNAs (miRNAs), as blood biochemical markers, have recently received more attention (Vijayan and Reddy, 2016; Martinez and Peplow, 2017; Vasudeva and Munshi, 2020). Increasing evidence suggests that miRNAs such as circulating miR-125a-5p, miR-125b-5p, and miR-143-3p are involved in CI (Tiedt et al., 2017). In addition, miRNAs have been reported as important regulators of synaptic plasticity in Alzheimer’s disease (Kou et al., 2020; Reza-Zaldivar et al., 2020) and neuronal development (Schratt et al., 2006; Jasinska et al., 2016). However, the role and function of circulating miRNAs in neuronal morphology after stroke remain to be resolved.

Here, we analyze miRNA expression profiling datasets of CI obtained from the Gene Expression Omnibus (GEO) DataSets portal and determine that hsa-miR-130a-3p is downregulated in CI *in vitro*. We demonstrate that miR-130a-3p regulates neuronal morphology and spinogenesis. We further show that miR-130a-3p protects neurons from damage induced by OGD/R treatment by targeting APP. Our results thus reveal a potential biochemical marker and mechanistic target for CI.

## Materials and Methods

### miRNA-seq Data From the GEO Database

**FIGURE 1:**
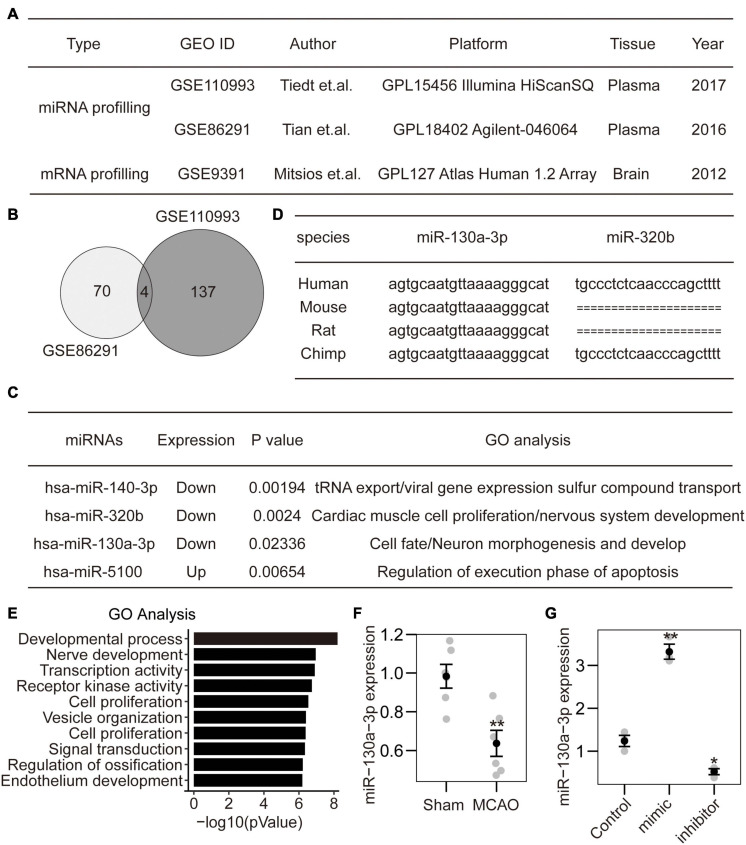
Identification of miRNA-130a-3p from CI expression profiles. **(A)** Two miRNA datasets and 1 mRNA dataset were selected from PubMed GEO datasets. **(B)** The intersection of two miRNA datasets included four miRNAs. **(C)** Among the identified miRNAs, three were downregulated, and one was upregulated. The GO analysis of target genes indicated that hsa-miR-320b and hsa-miR-130a-3p may play roles in nervous system development. **(D)** The sequence of hsa-miR-320b was not found in rodents. In contrast, the sequence of hsa-miR-130a-3p was conserved in both primates and rodents. **(E)** The detailed GO analysis indicated that hsa-miR-130a-3p mainly participated in the negative regulation of the developmental process and nerve development. **(F)** The expression of mmu-miR-130a-3p was downregulated in mouse brains after MCAO. **(G)** The expression of miR-130a-3p can be influenced by miR-130a-3p mimics and inhibitor in cultured neurons.

To identify differentially expressed miRNAs after CI, GEO datasets were analyzed. MiRNA datasets containing peripheral blood cells were excluded for the purpose of ruling out the effects of peripheral blood cells. Three GEO datasets were included, as shown in Figure 1A. Microarray profiles (up to 11 January 2018) related to CI were obtained from the GEO database^1^ with the following search strategy: (miR OR miRNA OR microRNA OR RNA-seq) AND (stroke OR ischemia OR hypoperfusion) AND (circulating OR plasma OR blood for microRNA datasets and [(mRNA(Title) OR RNA-seq(Title) OR gene(Title))] AND [stroke(Title) OR ischemia(Title) OR hypoperfusion(Title)] AND [brain(Title) OR cerebrum(Title)] for mRNA datasets. Five mRNA series were identified. The microarray data or RNA-seq data that met the following criteria were collected: (a) samples were from *Homo sapiens* and (b) examination of miRNA expression in circulating plasma or mRNA expression in the brain. Microarrays that did not provide useful data for analysis were excluded. Two miRNA expression profiles (GSE110993 and GSE86291) and 1 gene expression profile (GSE9391) were retrieved from the GEO database.

### Identification of DEGs and DE miRNAs

GSE110993 was analyzed using deseq2. GSE86291 was analyzed using GEO2R. The genes and miRNAs with fold change>1.5 and *p*-value < 0.05 were considered differentially expressed. The adjusted *p*-value (False Discovery Rate corrected *p*-value) for selection of DEGs and DE miRNAs was set as<0.05.

The target genes of the DEMs from GSE110993 and GSE86291 were predicted by the Cytargets plugin of Cytoscape, which is commonly used for predicting miRNA targets. The target genes were superposed with the DEGs to obtain an intersection dataset for further analysis.

### Functional and Pathway Enrichment Analyses

Gene Otology (GO) is widely used to annotate genes, gene products, and sequences. The Kyoto Encyclopedia of Genes and Genomes (KEGG) is a comprehensive database for biological interpretation of genome sequences and other high-throughput data. Both analyses are available in the Database for Annotation, Visualization, and Integrated Discovery database (DAVID database), which is a bioinformatics data resource composed of an integrated biological knowledge base and analytical tools to extract meaningful biological information from a large number of gene and protein datasets. Herein, GO and KEGG analyses were applied by using the DAVID database to identify DEGs. The cut-off criterion was set as *p*-value < 0.05.

### Cell Culture

Neuro2A cells were provided by Stem Cell Bank, Chinese Academy of Sciences (Shanghai, China), and maintained in DMEM supplemented with 10% FBS and 1% penicillin/streptomycin mix. Cells were routinely grown in a humidified atmosphere containing 95% air and 5% CO2 at 37°C.

Primary cell culture of hippocampal neurons was performed as described (Xu and Henkemeyer, 2009). Briefly, hippocampal neurons were dissociated from postnatal day 0 (P0) pups. The triturated cells (1 × 10^5^ cells per well) were grown on glass coverslips coated with 10 μM polylysine overnight in 24-well dishes. Then, the culture was grown in neurobasal A medium (Gibco, Thermo Fisher Scientific Inc.) supplemented with B27 and 2 mM glutamine. On day 3, the neurons were transfected with plasmids using the calcium phosphate method and cultured for the indicated number of days. Then, neurons were fixed (4% paraformaldehyde and 4% sucrose in PBS) and imaged to analyze the dendritic branches and spines. ImageJ software was used for Sholl analysis. To score the shape of neuronal spines, we used the NeuronStudio software package and an algorithm with the following cut off values: AR_thin (crit) = 2.5, HNR (crit) = 1.3, and HD (crit) = 0.4 μm. Protrusions with lengths of 0.2–3.0 μm and max widths of 3 μm were counted. Spine density was calculated by dividing the total spine number by the dendritic branch length.

### miRNA Mimics and Inhibitor, Cell Transfection

miR-130a-3p mimics, inhibitor, and scramble control were designed and synthesized by HuaGene (Shanghai, China), and the detailed sequences are shown in Table 1. The cDNA fragment of the APP open reading frame was inserted into the pcDNA3.1 vector to generate the *APP* expression vector. The construct was transfected into cells using Lipofectamine 2000 transfection reagent (Invitrogen, Carlsbad, CA, United States).

**TABLE 1:** Sequences of miR-130a-3p mimics, inhibitor and scramble control.

	**Mimic (5**′**–3**′)	**Inhibitor (5**′**–3**′)
miR-130a-3p	AGUGCAAUGUUAAA AGGGCAU	AUGCCCUUUUAACA UUGCACU
scramble control	GUUAAGGAUCAGCG AGAAUAU	

### RNA Isolation and Quantitation

Total RNA was extracted using the trizol reagent (Invitrogen TM) according to the instructions. Primer sequences for APP were 5′-GTCCCTGCTCTACAATGTCC-3′ (forward) and 5′-CTTCACTTCCGAGATCTCTTCC-3′ (reverse); and for glyceraldehyde-3-phosphate dehydrogenase (GAPDH) were 5′-AATGGTGAAGGTCGGTGTG-3′(forward) and 5′- GTGGAGTCATACTGGAACATGTAG-3′ (reverse); and for miR-13a-3p was 5′-AGUGCAAUGUUAAAAGGGCAU-3′. The reverse primer of miRNA and the forward primer of U6 were provided by the Mir-X miRNA First-Strand Synthesis and TB Green qRT-PCR Kit (Takara Bio).

Total miRNA was reverse-transcribed into complementary DNA (cDNA) according to the instructions of Mir-X miRNA First-Strand Synthesis (Takara Bio). And total mRNA was reverse-transcribed into cDNA with Fast Quant RT Kit (TianGEN). With cDNA as a template, RT-qPCR was performed on an ABI 7500 instrument (Applied Biosystems) for miRNA using TB Green qRT-PCR Kit (Takara Bio), and for mRNA using Fast SYBR Green Master Mix (Applied Biosystems). With U6 and GAPDH serving as the loading control, the expression ratio of the target miRNA and gene between the experimental and control groups was calculated using the 2^–ΔΔ*Ct*^ method.

### Dual-Luciferase Reporter Assay

The wild-type *APP* 3′-UTR sequence was obtained from PubMed gene datasets. The mutant *APP* 3′-UTR sequence was generated by deleting the target sequence ATCGCCTTTTGACAGCTGTGCTG predicted by bioinformatic analysis. The wild-type fragment containing the predicted binding site of miR-130a-3p or mutant *APP* 3′-UTR was inserted into the pMIR-REPORT Luciferase H306 vector (OBiO Technology (Shanghai) Corp., Ltd.). HEK 293 cells were cotransfected with a pMIR-REPORT vector containing the wild-type or mutant *APP* 3′-UTR and miR-130a-3p mimics or inhibitor using Lipofectamine 2000 (Invitrogen). After culturing for 48 h, cells were lysed, and the luciferase activity was measured using the Dual-Luciferase Reporter Assay System according to the manufacturer’s protocol (Promega). For each transfected cell sample, the firefly luciferase activity was normalized to Renilla luciferase activity.

### Induction of the OGD/R Model

Primary neurons were cultured for 24 h under normal conditions (95% air and 5% CO2) at 37°C. Then, the cells were placed in glucose-free DMEM and maintained under hypoxic conditions (3% O2/5% CO2/92% N2) for 8 h. Afterward, the medium was discarded, and fresh medium containing glucose was added. Then, cells were cultured under normal conditions for another 24 h. Neurons maintained in normal medium and normal conditions were used as controls.

### Mouse Model

Eight-week-old male C57BL/6 mice (purchased from Jiesijie Lab Animal Ltd, Shanghai, China). The animal study was reviewed and approved by Animal Care and Use Committee of Shanghai Medical College of Fudan University. The mouse MCAO model was established as described previously (Song et al., 2018) to induce ischemic lesions. In brief, mice were anesthetized with intraperitoneal injection of 1% pentobarbital sodium (35 mg/kg). A thermostatically controlled heating pad was utilized to maintain rectal temperature at 37°C. An intraluminal filament (Guangzhou Jialing Biotechnology Co., Ltd., China) with a 0.105-mm-diameter body and a 0.2-mm-diameter tip was inserted into the internal carotid artery through the severed external carotid artery to obstruct MCA blood flow for 30 min. Laser Doppler flowmetry (VMS-LDF2; Moor Instruments Ltd, United Kingdom) was performed to monitor regional cerebral blood flow. This study excluded mice with less than 20% reduction in cerebral blood flow in the core area of the MCA area. Animals were sacrificed after 24 h of reperfusion.

### Western Blot Analysis

Protein lysates from cultured cells were prepared with RIPA lysis buffer containing a protease and phosphatase inhibitor cocktail (Sigma-Aldrich, St. Louis, MO, United States). Equal amounts of protein lysates were resolved by 10% SDS-polyacrylamide gel electrophoresis. Then, the separated proteins were transferred onto a PVDF membrane followed by incubation with 5% nonfat milk powder at 37°C for 1 h. The membrane was then incubated with primary and respective secondary antibodies. Primary antibody against *APP* was purchased from Santa Cruz Biotechnology, and the GAPDH antibody was purchased from Abcam (Cambridge, MA, United States). Protein bands were visualized using the ECL western blotting detection system (Thermo Fisher Scientific, Inc., Waltham, MA, United States).

### Statistical Analysis

Data are presented as the mean ± standard deviation. Continuous variables were reported as mean (standard deviation) were compared using Student’s *t*-test or one-way or two-way ANOVA, as appropriate. Data were processed using SPSS Statistics Version 22.0 (SPSS Inc., Chicago, IL, United States). Significance was set at a *p*-value less than 0.05.

## Results

### Identification of miRNA-130a-3p From CI Expression Profiles

To identify specific miRNA diagnostic biomarkers and potential mechanisms for CI, we analyzed expression profiling datasets of CI stroke obtained from the Gene Expression Omnibus (GEO) DataSets portal. According to the inclusion criteria, 2 miRNA expression profiling datasets (GSE110993 and GSE86291), and 1 mRNA expression profiling dataset GSE9391 were obtained (Figure 1A). After normalization of the original miRNA (with DESeq2 package) and mRNA expression data (with quantile methods), we performed differentially expressed analysis between ischemic stroke and normal control samples. Finally, a total of 70 and 137 miRNAs were considered as significantly differentially expressed with *p*-value less than 0.05 in GSE110993 and GSE86291, respectively. Four miRNAs were identified in both miRNA datasets (Figure 1B), of which 1 was upregulated and 3 were downregulated (Figure 1C). Among them, hsa-miR-320b and hsa-miR-130a-3p were mainly related to nervous system development and neuronal morphogenesis (Figure 1C). Furthermore, hsa-miR-130a-3p was conserved among different species and is expressed in humans, rodents, and primates (Figure 1D). The GO analysis showed that hsa-miR-130a-3p mainly participated in the negative regulation of the development process, nerve development and transcription coregulator activity (Figure 1E). Thus, hsa-miR-130a-3p was potentially more important during CI stroke than hsa-miR-320b. To further confirm the predicted results, we established the MCAO model in mice (6 vs. 6) and found that the expression of mmu-miR-130a-3p in brain tissue was significantly lower in the MCAO group than in the sham group (Figure 1F). These data suggest that miR-130a-3p may be a potential biochemical marker for CI stroke and may be involved in the regulation of neuronal development.

### miRNA-130a-3p Mediates Neuronal Morphogenesis and Spinogenesis

**FIGURE 2:**
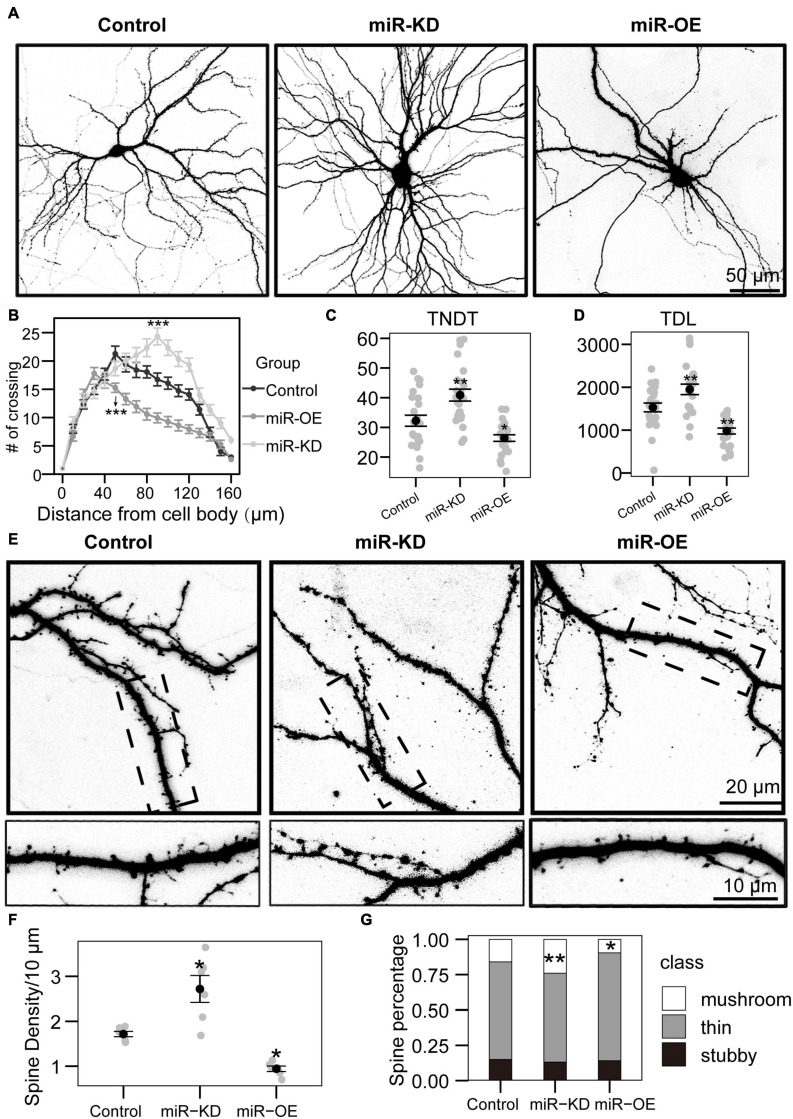
miRNA-130a-3p mediates the branching of dendrites and the growth of dendritic spines. **(A)** Representative images of hippocampal neurons transfected on DIV7 for 7 days with control, miRNA-130a-3p-OE or 130a-3p-inhibitor. **(B)** Sholl analysis of neurons transfected with control, miRNA-130a-3p-OE or 130a-3p-inhibitor (control: *n* = 24; miRNA-130a-3p-OE: *n* = 24; 130a-3p-inhibitor: *n* = 24). **(C,D)** TNDT and TDL of neurons transfected with Control, miRNA-130a-3p-OE or 130a-3p-inhibitor (Control: *n* = 24; miRNA-130a-3p-OE: *n* = 24; 130a-3p-inhibitor: *n* = 24), respectively. **(E)** Representative images of hippocampal neurons transfected on DIV7 for 14 days with control, miRNA-130a-3p-OE or 130a-3p-inhibitor. **(F,G)** Quantification of dendritic spine densities and the percentages of classification of neurons in **(E)** (control: *n* = 24; miRNA-130a-3p-OE: *n* = 24; 130a-3p-inhibitor: *n* = 24), respectively. Cell images were obtained from three independent culture batches. Error bars indicate S.E. ***p-value < 0.001; **p-value < 0.01; *p-value < 0.05; *miR*-KD, miR-130a-3p knockdown; miR-OE, miR-130a-3p overexpression.

To investigate the function of miR-130a-3p in dendrite growth, a specific miR-130a-3p inhibitor and control plasmids expressing enhanced green fluorescence protein (GFP) were used. And they can influence the expression of miR-130a-3p (Figure 1G). To quantify neuronal complexity, Sholl analysis, a common analytical method for assessing neuronal dendrite branches by quantifying the number of dendrites crossing the circle at different radial distances from the cell soma (Sholl, 1953), was used. In the control group, the number of crossings increased, reaching a maximum at 50 μm, and gradually declined until the last measurement point (160 μm from the cell body) (Figures 2A,B). Neurons treated with the miRNA-130a-3p inhibitor showed an obvious change as the number of crossings reached a peak at 80 μm from the cell soma and were still much higher than that in control neurons at 160 μm. We also found that transfection with the miRNA-130a-3p inhibitor increased the total number of dendritic tips (TNDT) by approximately 27% (40.9 vs. 32.2, *p* < 0.01), and led to a strong increase in total dendrite length (TDL) by approximately 28% compared with the control (1949.5 vs. 1528.8, *p* < 0.01) (Figures 2C,D). These results indicate that downregulation of miRNA-130a-3p promotes neurite outgrowth.

As inhibition of miR-130a-3p in hippocampal neurons promotes the branching of dendrites, we speculate that miR-130a-3p plays a negative role in the process of neuronal dendritic growth. To test this hypothesis, we overexpressed miRNA-130a-3p in cultured neurons. As shown in Figures 2A,B, Sholl analysis showed that the number of crossings reached a maximum at 30 μm in the miRNA-130a-3p-OE group, which differed significantly from the control, and the “peak” of branching was shifted leftward (closer to the soma) (Figures 2A,B). In addition, we found that overexpression of miRNA-130a-3p significantly decreased the TNDT and TDL of neurons (Figures 2A,C,D). The miRNA-130a-3p-OE neurons showed an ∼20% decrease in TNDT (26.4 vs. 32.2, *p* < 0.05) and an ∼36% decrease in TDL (978.2 vs. 1528.8, *p* < 0.01) compared with the control.

In addition to its effect on overall dendritic morphology, miRNA-130a-3p also played a role in regulating dendritic spines. As shown in Figures 2E–G, we found that knockdown of miRNA-130a-3p resulted in a significant increase in the density of dendritic spines by 58% compared with the control (2.7 vs. 1.7, *p* < 0.05), while overexpression of miRNA-130a-3p showed the opposite phenotype, with a 46% reduced density of dendritic spines (0.9 vs. 1.7, *p* < 0.05) (Figure 2F). We also found that neurons treated with the miRNA-130a-3p inhibitor exhibited an increase in the ratio of mushroom-shaped spines with a concomitant obvious reduction in the ratio of filopodia/thin-like protrusions, while the opposite effect was observed in the miRNA-130a-3p overexpression group. Dendritic spines in the control group were composed of 69.1% thin/filopodia, 14.9% stubby, and 15.9% mushroom spines, whereas in the miRNA-130a-3p inhibitor group, there was an increase in mushroom spines by 8.0% (*p* < 0.01), with a reduction in thin/filopodia-like and stubby spines by 6.2 and 1.8%, respectively, while in the miRNA-130a-3p-OE group, there was a decrease in mushroom spines by 6.4% (*p* < 0.05) and an increase in thin/filopodia-like and stubby spines by 7.2 and −0.8%, respectively, (Figure 2G). These data suggest that miRNA-130a-3p is necessary and sufficient for regulating neuronal morphogenesis and spinogenesis.

### APP Is the Target Gene of miR-130a-3p

To explore the mechanism of miR-130a-3p-mediated neurite outgrowth, miR-130a-3p targets were searched by computer-aided miRNA target prediction programs, which include the TargetScan, miRBase, and miRBase databases, and 1234 genes were identified. Then, we combined the DEG, CytoHubba, and the miRNA-gene network data to further identify reliable hub genes. Eight hub genes, *APP, CREB5, TNFSF10, RPS6KA3, SNAP25, PDGFRA, ATP2B2*, and *ITGA4*, were screened. APP, a well-known gene involved in neurodegenerative diseases such as Alzheimer’s disease, has been implicated as a regulator of synapse formation and neural plasticity (Zhu et al., 2017). Thus, we were particularly interested in whether miR-130a-3p mediated the growth of dendritic branches and spines through the *APP* gene.

**FIGURE 3:**
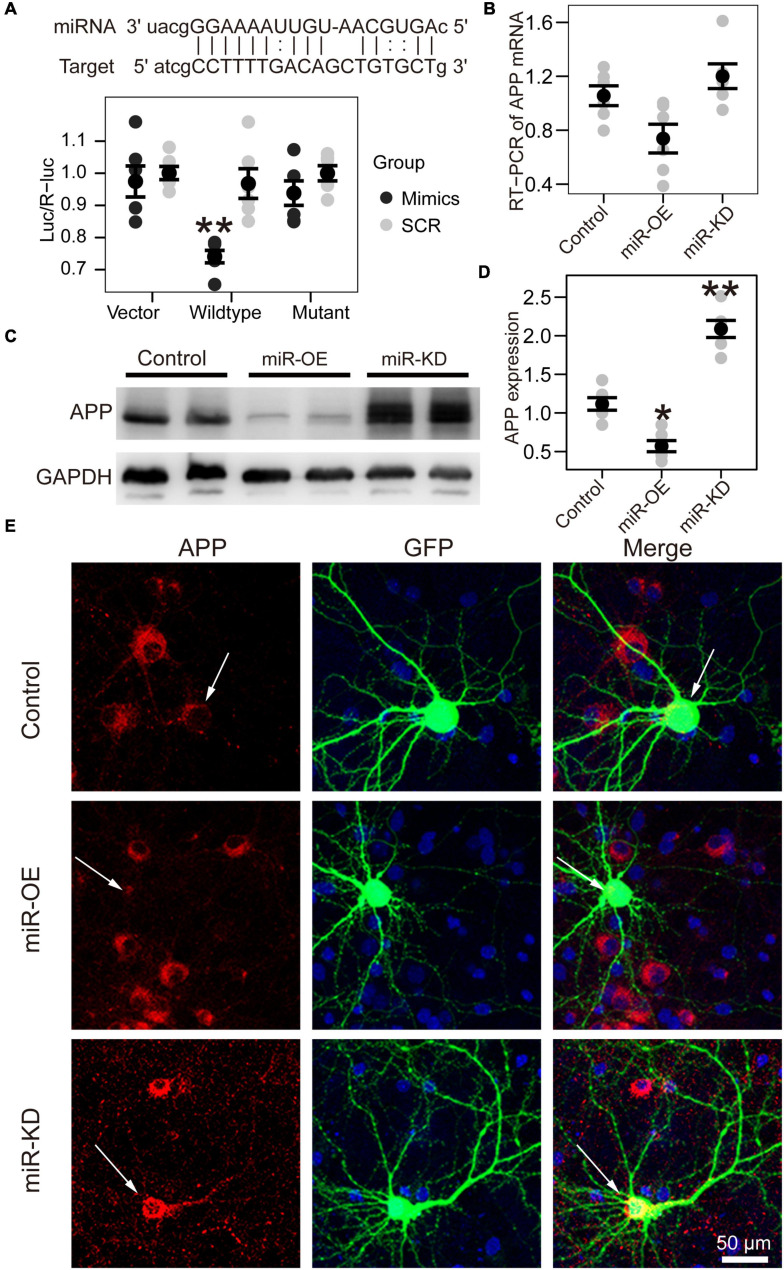
APP is the target gene of miR-130a-3p. **(A)** Up: The putative target gene sequence of *APP* mRNA 3′ UTR and miR-130a-3p sequence. Down: Results of dual luciferase reporter assay. **(B)** Real-time reverse transcription PCR (RT-PCR) data for *APP* mRNA in the Neuro2A cell line transfected with reagents for the overexpression and inhibition of miR-130a-3p. Data were normalized to GAPDH mRNA. **(C,D)** Western blot of *APP* protein in Neuro2A cell line transfected with reagents for the overexpression and inhibition of miR-130a-3p. **(E)** Immunofluorescence experiment of *APP* in cultured mouse hippocampal neurons transfected with reagents for the overexpression and inhibition of miR-130a-3p. The arrow represents transfected neurons. *P < 0.05, **P < 0.01 versus the control group; *miR*-KD, miR-130a-3p knockdown; miR-OE, miR-130a-3p overexpression.

Bioinformatic analysis predicted that miR-130a-3p can target the 3′-UTR of human APP. To confirm that miR-130a-3p targeted the 3′-UTR of APP, we performed dual-luciferase reporter assays and found that overexpression of miR-130a-3p significantly decreased the luciferase activity of the reporter vector containing the wild-type *APP* 3′-UTR (*p* < 0.01) (Figure 3A). As a control, we deleted the target sequence of miR-130-3p to mutate the 3′-UTR of human *APP* and found that miR-130a-3p showed no significant effect on the luciferase activity of the reporter vector containing the mutant 3′-UTR (Figure 3A). We then asked whether *APP* was regulated by miR-130a-3p, and we investigated the effect of miR-130a-3p on *APP* expression by RT-PCR, but no significant change was found (Figure 3B). To further confirm that the expression of *APP* protein in neurons was controlled by mmu-miR-130a-3p, we transfected the control plasmid and mmu-miR-130a-3p mimics and inhibitor plasmids into primary hippocampal neurons from APP/PS1 mice. Three days after transfection, we found a significant reduction (*p* < 0.05) in *APP* expression in the mimics group compared with the control (Figures 3C,D). Conversely, inhibition of miR-130a-3p increased *APP* expression (*p* < 0.01) (Figures 3C,D), which was also confirmed by immunostaining (Figure 3E). Collectively, these results suggested that miR-130a-3p regulated the expression of *APP* in neurons.

### miR-130a-3p Can Rescue Spine Loss After OGD Treatment

**FIGURE 4:**
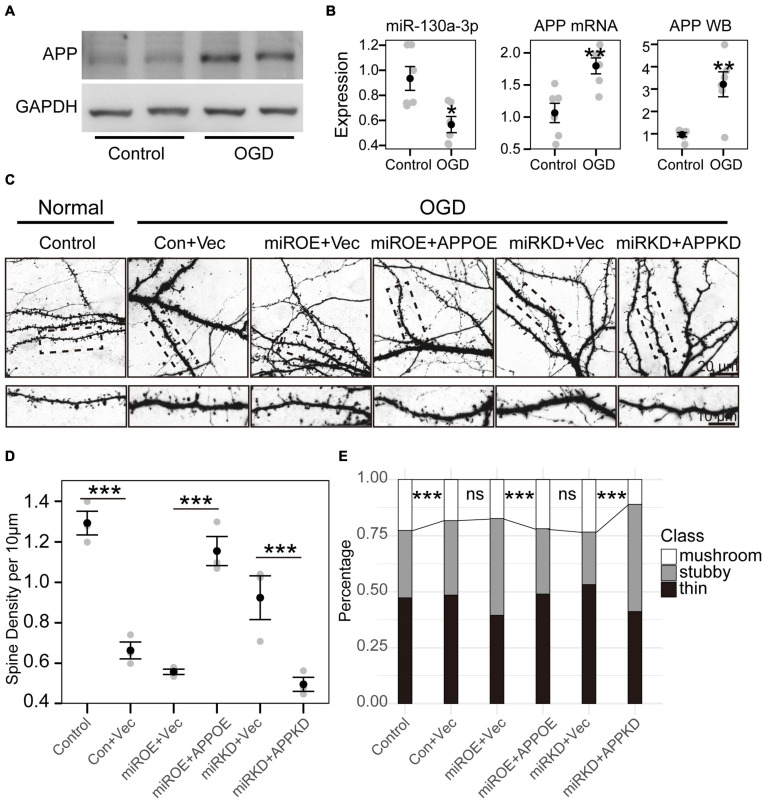
miR-130a-3p can rescue spine loss after OGD treatment. **(A)** Western blot of *APP* protein in cultured neurons treated with control or OGD/R. **(B)** OGD/R reduced miR-130a-3p expression, but increased the expression of APP mRNA and APP protein. **(C)** OGD/R significantly reduced spine density, while overexpression of miR-130a-3p further decreased spine density. In contrast, downregulation of miR-130a-3p in OGD/R-treated neurons partially increased spine density. Downregulation of *APP* partially reversed the promoting effect of miR-130a-3p inhibition on the density of total and mushroom-shaped spines. The overexpression of *APP* significantly reversed the spine loss induced by miR-130a-3p overexpression in neurons treated with OGD/R. **(D,E)** The statistical results of the OGD/R experiment. *P < 0.05, **P < 0.01, ***P < 0.001 versus the control group; Con, Control; Vec, vector; miRKD, miR-130a-3p knockdown; miROE, miR-130a-3p overexpression; APPOE, *APP* overexpression; APPKD, *APP* knockdown.

To investigate the precise biological effect of miR-130a-3p in regulating OGD/R-induced neuronal injury, we treated these induced neurons with miR-130a-3p mimics or miR-130a-3p inhibitor to observe spine morphology. We firstly detected endogenous miR-130a-3p and APP changes (Figure 4A) after OGD. The expression of miR-130a-3p was reduced (*p* < 0.05), but APP mRNA and APP protein were increased (*p* < 0.01) (Figure 4B). Consistent with previous studies on CI (Zhang et al., 2005; Brown et al., 2008), OGD/R-treated neurons showed significantly reduced spine density (*p* < 0.001) with loss of mature, mushroom-shaped spines, while overexpression of miR-130a-3p further decreased spine density (no significance) (Figures 4C–E). Conversely, downregulation of miR-130a-3p in OGD/R-treated neurons had the opposite effect: the total and mushroom-shaped spine density increased (no significance) (Figures 4C–E). These results suggest that suppression of miR-130a-3p may exert a neuroprotective effect in OGD/R-treated neurons.

To further confirm whether miR-130a-3p inhibition alleviated OGD/R-induced neuronal injury by upregulating APP, we detected the effect of *APP* knockdown on the miR-130a-3p inhibition-induced protective effect. We found that transfection of *APP* shRNA partially reversed the promoting effect of miR-130a-3p inhibition on the density of total and mushroom-shaped spines (*p* < 0.001) (Figures 4C–E). As expected, the overexpression of *APP* significantly reversed the spine loss induced by miR-130a-3p overexpression in neurons with OGD/R treatment (*p* < 0.001). Taken together, these results suggest that downregulation of miR-130a-3p attenuates OGD/R-induced spine loss by upregulating APP.

## Discussion

Cerebral ischemia brings worldwide burden (Donkor, 2018). Although many efforts have been made, the therapeutic effect is still insufficient (Thompson and Ronaldson, 2014). The detailed mechanism is still not fully understood. MiRNAs may play important roles in this process (Ouyang et al., 2013; Wang et al., 2013). Accordingly, our goal was to characterize early miRNA changes in blood plasma and investigate its influence on neurons. Our results indicated that miR-130a-3p expression was downregulated in blood plasma following ischemic stroke. We further demonstrated that inhibition of miR-130a-3p promoted *APP* expression, which decreased OGD/R-induced spine loss. Our study suggested that the miR-130a-3p/*APP* axis played an important role in regulating OGD/R-induced neuronal injury, implying a potential role of the miR-130a-3p/*APP* axis in the pathogenesis of CI/reperfusion injury.

Accumulating evidence suggests that miRNAs are excellent biomarkers for ischemic stroke (Sonoda et al., 2019; Kalani et al., 2020). In the present study, we sought to determine the effects of miRNAs by integrated analysis of miRNA and mRNA expression profiles obtained from the GEO database. To remove the effect of circulating blood cells, only the miRNA datasets of plasma were chosen. Based on the results from miRNA analysis, 4 differentially expressed miRNAs were selected. The GO analysis of the target genes indicated that miR-130a-3p and miR-320b might play roles in the nervous system. To further explore the function of the two miRNAs in cultured mouse neurons, miRNA conservation across different species was investigated, which showed that the sequence of miR-130a-3p is conserved among humans, primates, and rodents, whereas miR-320b was not found in rodents. In addition, the expression of miR-130a-3p was lower in the blood plasma of mice after MCAO. Therefore, miR-130a-3p was selected for further investigation of its function during cerebral stroke.

Increasing numbers of studies have shown that miR-130a-3p is downregulated after stroke and may be an important regulator of brain injury (Wang et al., 2018; Zheng et al., 2019), but its detailed mechanism has rarely been investigated. Indeed, the GO analysis of miR-130a-3p indicated that it mainly participates in the negative regulation of development and nerve development. Therefore, we speculated that miR-130a-3p may exert an influence on neuronal function during CI.

To verify this speculation, we tested the effect of miR-130a-3p on neurons *in vitro*. Our data showed that following the downregulation of mmu-miR-130a-3p, the complexity of hippocampal neuron dendrites, TNDT, and TDL was enhanced, whereas overexpression of mmu-miR-130a-3p had the opposite effect. In addition to dendrites, dendritic spines are also important for neuronal functions (Stein and Zito, 2019). The downregulated expression of mmu-miR-130a-3p enhanced neuronal spine density and the ratio of mushroom-type dendritic spines, which represents the mature status of spines. In contrast, overexpression of mmu-miR-130a-3p decreased neuronal spine density and the ratio of mushroom-type dendritic spines. Overall, mmu-miR-130a-3p promoted the outgrowth of neuron dendrites and the maturation of dendritic spines.

We then asked how mmu-miR-130a-3p affected neuronal morphology. Most studies have hitherto revealed that miRNAs function by binding to a specific sequence at the 3′ UTR of their target mRNAs to induce degradation and translational repression of mRNAs (O’Brien et al., 2018). A previous study showed that in sensory dorsal root ganglia, VEGFR-2 expression increased during maturation and was accompanied by an overexpression of miR-130a-3p, which indicates that miR-130a-3p may affect neuron outgrowth through VEGFR-2 (Glaesel et al., 2020). However, VEGFR-2 was not significantly changed in the selected mRNA datasets of stroke brain tissue. For this reason, miR-130a-3p did not affect neurons through VEGFR-2 after stroke. The potential target genes were obtained from the overlap between the predicted target genes and the differentially expressed mRNAs in the stroke brain. Among them, *APP* is a well-known gene involved in neurodegenerative diseases (Whalley, 2009; Pluta et al., 2020), whose 3′-UTR contains a target sequence of miR-130a-3p. We found that mmu-miR-130a-3p can impact the expression of *APP* by binding to the 3′-UTR of *APP* by utilizing a dual luciferase experiment. We further analyzed its impact on *APP* mRNA by RT-PCR, and no significant change was found. This result may be attributed to the incomplete complementarity of mmu-miR-130a-3p and its target sequence. Furthermore, the protein expression of *APP* was examined. Both western blotting and immunofluorescence indicated that overexpression of miR-130a-3p decreased the expression of *APP* protein. In contrast, downregulation of miR-130a-3p had the opposite effect. We further discovered that miR-130a-3p can rescue OGD-induced dendritic spine loss and spine immature through APP. However, the precise mechanism for dendritic spine changes associated with *APP* is still not clear. c-Jun N-terminal kinase (JNK), JNK interacting protein, Fe65, D-serine, etc., may play important roles in this process (Montagna et al., 2017). Overall, miR-130a-3p may function in the nervous system by targeting *APP* to regulate neuronal function in physiological and pathological conditions.

In conclusion, our findings indicate that the downregulation of miR-130a-3p can protect neurons from OGD-induced damage by targeting APP. Further work examining miR-130a-3p functionality in ischemic animals is merited.

## Data Availability Statement

The datasets presented in this study can be found in online repositories. The names of the repository/repositories and accession number(s) can be found below: https://www.ncbi.nlm.nih.gov/geo/, GSE110993. https://www.ncbi.nlm.nih.gov/geo/, GSE86291, and https://www.ncbi.nlm.nih.gov/geo/, GSE9391.

## Ethics Statement

The animal study was reviewed and approved by Animal Care and Use Committee of Shanghai Medical College of Fudan University.

## Author Contributions

LiZ and BY designed the research. LiZ performed the research, analyzed the data, and wrote the manuscript. LeZ and JT helped to design the experiment. KC cultured the mouse neuron. All authors contributed to the article and approved the submitted version.

## Conflict of Interest

The authors declare that the research was conducted in the absence of any commercial or financial relationships that could be construed as a potential conflict of interest.

## Publisher’s Note

All claims expressed in this article are solely those of the authors and do not necessarily represent those of their affiliated organizations, or those of the publisher, the editors and the reviewers. Any product that may be evaluated in this article, or claim that may be made by its manufacturer, is not guaranteed or endorsed by the publisher.

## Footnotes

1
http://www.ncbi.nlm.nih.gov/geo/
